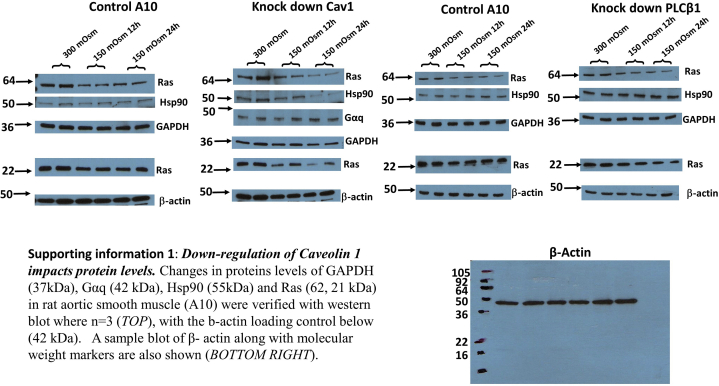# Deformation of caveolae impacts global transcription and translation processes through relocalization of cavin-1

**DOI:** 10.1016/j.jbc.2022.102005

**Published:** 2022-05-02

**Authors:** Androniqi Qifti, Shravani Balaji, Suzanne Scarlata

**Affiliations:** Department of Chemistry and Biochemistry, Worcester Polytechnic Institute, Worcester, Massachusetts, USA

**Keywords:** cavin-1, nuclear relocalization, cell stress, caveola domains, protein translation, DLS, dynamic light scattering, HBSS, Hank’s Balanced Salt Solution, MEF, mouse embryonic fibroblast, N&B, number and brightness, p-bodies, processing bodies, PABPC1, polyadenylate-binding protein (cytosolic)-1, RISC, RNA-induced silencing complex

## Abstract

Caveolae are invaginated membrane domains that provide mechanical strength to cells in addition to being focal points for the localization of signaling molecules. Caveolae are formed through the aggregation of caveolin-1 or -3 (Cav1/3), membrane proteins that assemble into multifunctional complexes with the help of caveola-associated protein cavin-1. In addition to its role in the formation of caveolae, cavin-1, also called polymerase I and transcript release factor, is further known to promote ribosomal RNA transcription in the nucleus. However, the mechanistic link between these functions is not clear. Here, we found that deforming caveolae by subjecting cells to mild osmotic stress (150–300 mOsm) changes levels of GAPDH, Hsp90, and Ras only when Cav1/cavin-1 levels are reduced, suggesting a link between caveola deformation and global protein expression. We show that this link may be due to relocalization of cavin-1 to the nucleus upon caveola deformation. Cavin-1 relocalization is also seen when Cav1-Gαq contacts change upon stimulation. Furthermore, Cav1 and cavin-1 levels have been shown to have profound effects on cytosolic RNA levels, which in turn impact the ability of cells to form stress granules and RNA-processing bodies (p-bodies) which sequester and degrade mRNAs, respectively. Our studies here using a cavin-1-knockout cell line indicate adaptive changes in cytosolic RNA levels but a reduced ability to form stress granules. Taken together, our findings suggest that caveolae, through release of cavin-1, communicate extracellular cues to the cell interior to impact transcriptional and translational.

Caveolae are flask-shaped membrane invaginations that can flatten to provide more membrane area and are implicated in mechanosensation, electric sensation, endocytosis, and vasodilation through modulating the NO pathway (1, 2, 3, 4). In previous studies, our lab found that populations of Gαq and their receptors reside in caveola domains and this localization is assisted by interactions between Gαq and caveolin molecules (5, 6). Activation of Gαq by hormones or neurotransmitters strengthens these interactions resulting in enhancement of calcium signals. Deformation of caveolae by mild osmotic stress disrupts this stabilization and returns calcium signals to levels observed in the absence of caveolae (7, 8, 9). Additionally, when cells are subjected to either bidirectional static or oscillating mechanical stretch, calcium release through activation of Gαq/PLCβ is intact, but contacts between Gαq and caveolin are disrupted (10, 11). In separate series of studies, our lab found that activation of the Gαq/PLCβ triggers a novel calcium-independent pathway that is linked to regulation of GAPDH protein production but not Hsp90 (12).

Cavins are a family of four proteins that regulate the curvature of the caveola membrane by anchoring caveolins to the cytoskeleton (for reviews, refer to the studies by Briand et al (13), Kovtun et al (14), and Williams and Palmer (15)). The most abundantly expressed is cavin-1, also known as polymerase 1 and transcript release factor or cav-p60. Since its discovery in 1998, cavin-1 has been found to be a necessary component of caveola formation by mediating the sequestration of caveolin molecules into immobile caveola domains (refer to the study by Briand et al (13)). Several studies, including those here, strongly suggest that expression of cavin-1 and caveolin are interdependent; Cav1-knockout (KO) mice have nearly no cavin-1 expression, and cavin-1 KO mice have diminished Cav1 expression (15, 16). When fibroblasts are swelled by a 10-fold decrease in osmotic strength, cavin-1 is released from the plasma membrane as caveolae disassemble to provide more membrane area (17).

Before cavin-1 was identified as a structural adapter for caveolae, it was recognized for its role in modulating cellular transcriptional activity (18). Cavin-1/polymerase 1 and transcript release factor promotes ribosomal DNA transcription by binding to the 3′ pre-RNA, allowing the release of pre-RNA and Polymerase I from the transcription complex (19). Cavin-1 not only plays a role in transcript release but also increases the overall rate of transcription in a concentration-dependent manner. In adipocytes, insulin stimulation causes phosphorylation of cavin-1 promoting its translocation from caveolae to the nucleus (19) suggesting a role in signal transduction. The importance of cavin-1 expression is seen in KO mice that show diverse abnormalities consistent with impaired ribosome biogenesis including abnormal growth failure, loss in fat, resistance to obesity, impaired exercise ability, muscle hypertrophy, altered cardiac, and lung function (20). Pertinent for this study are reports suggesting that increased cavin-1 expression promotes cellular stress responses to toxic agents which may be traced to binding to p53 in the cytosol (21).

The connection between cavin-1’s ability to regulate caveola structure and promote ribosomal RNA production suggests that any mechanism that destabilizes cavin-1’s interactions with caveolin including caveola deformation would impact transcription and transitional processes through cavin-1. Here, we show that changes in environmental conditions, such as mild osmotic stress, addition of neurotransmitter, or exposure toxins, drive cavin-1 from the plasma membrane to impact RNA transcription and two processes that regulate protein translation (*i.e.*, stress granule and p-body formation). Our results suggest that cavin-1 molecules of caveolae act as sensors to inform the cell interior of environmental stress.

## Results

### Expression of Cav1 alters protein expression in response to stress

We have previously found that the binding of cytosolic PLCβ to C3PO, the promoter of the RNA-induced silencing complex (RISC), inhibits its activity to regulate the silencing of select genes and that this effect is reversed upon Gαq activation (12, 22, 23, 24). Specifically, we found that activation of Gαq drives cytosolic PLCβ to the plasma membrane releasing inhibition of the promoter of RISC and reversing silencing of GAPDH by siRNA, but not Hsp90. We tested the idea that because the caveola enhances activation of Gαq (5), which shifts the cytosolic population of PLCβ to the plasma membrane, then caveola expression could indirectly regulate GAPDH production.

**Figure 1:**
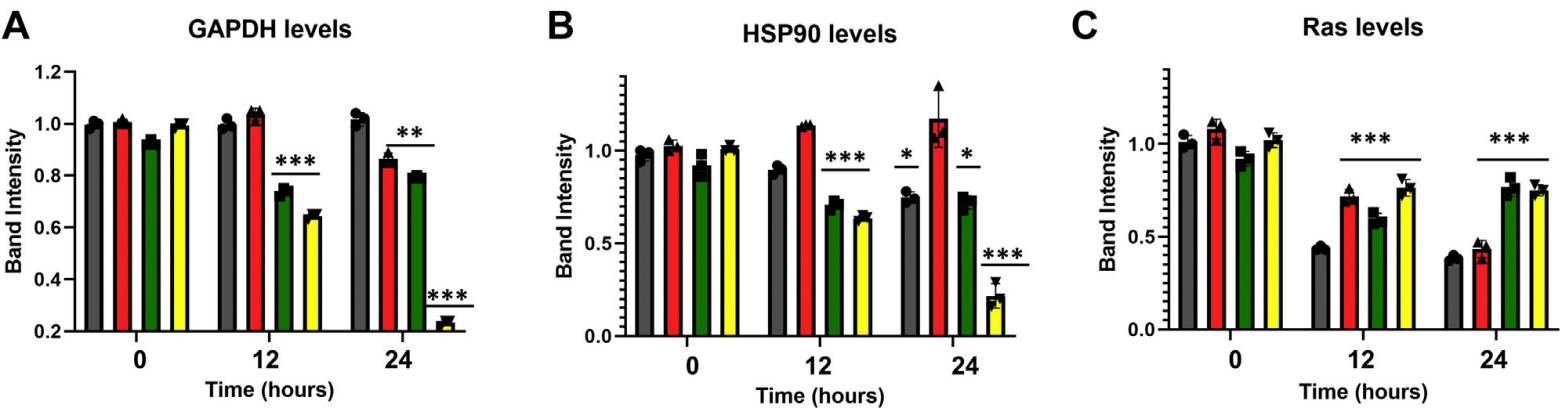
**Downregulation of Caveolin one impacts protein levels**. *A*–*C*, changes in proteins levels of (*A*) GAPDH, (*B*) Hsp90, and (*C*) Ras in rat aortic smooth muscle (A10) in control (*black*), siRNA PLC (*red*), siRNA Gαq (*green*), and siRNA Caveolin-1 (*yellow*) cells where n = 3 and the *asterisks* correspond to *p* values, with one *asterisk* being 0.1<*p* < 0.5, two *asterisks* meaning *p* < 0.05, and three *asterisks* indicating *p* < 0.001. An example Western blot is shown in Fig. S1.

Using rat aortic smooth muscle (A10) cells, we quantified the production of GAPDH, along with Hsp90 and Ras for comparison, when Cav1, Gαq, and PLCβ were downregulated. We first found that reducing Cav1 changes the level of actin and reduces the level of other cellular proteins (discussed in the later part of the article), making direct impact of Cav1 levels difficult to assess. We therefore took a more indirect approach. Because caveola provides mechanical strength to cells, we subjected cells to mild osmotic stress that will deform caveola and eliminate its stabilization of the Gαq/PLCβ pathways (8). We reduced the osmolarity of the media from 300 to 150 mOsm in control cells and cells treated with siRNA(Cav1) and quantified changes in GAPDH, Hsp90, and Ras levels (Fig. 1, where samples blots are shown in Fig. S1). Differences between the two cell groups were not immediate but appeared ∼12 h after continuous osmotic stress, suggesting that Cav1 impacts slower transcription and/or translation processes rather than more rapid degradation or other downregulation mechanisms. In contrast to Cav1, downregulating PLCβ1 or Gαq had little or no effect, arguing that the Gαq/PLCβ pathway is not the primary factor underlying changes in protein production. These data suggest that Cav1 levels impact the ability of cells to produce proteins under hypo-osmotic stress conditions.

### Cavin-1 shifts its cellular localization under osmotic stress

Expression of Cav1 and cavin-1 are interdependent ((16) and Fig. S2*A*), and so we tested whether the protein changes observed in Figure 1 might be due to cavin-1 levels since cavin-1 promotes transcription of ribosomal RNA (18). While previous studies found that a tenfold change in osmotic stress disassembles caveolae releasing cavin-1 from the plasma membrane (17), it is unclear whether release will occur at the mild, physiological stress conditions used here which deforms but not disassembles caveolae (9).

**Figure 2:**
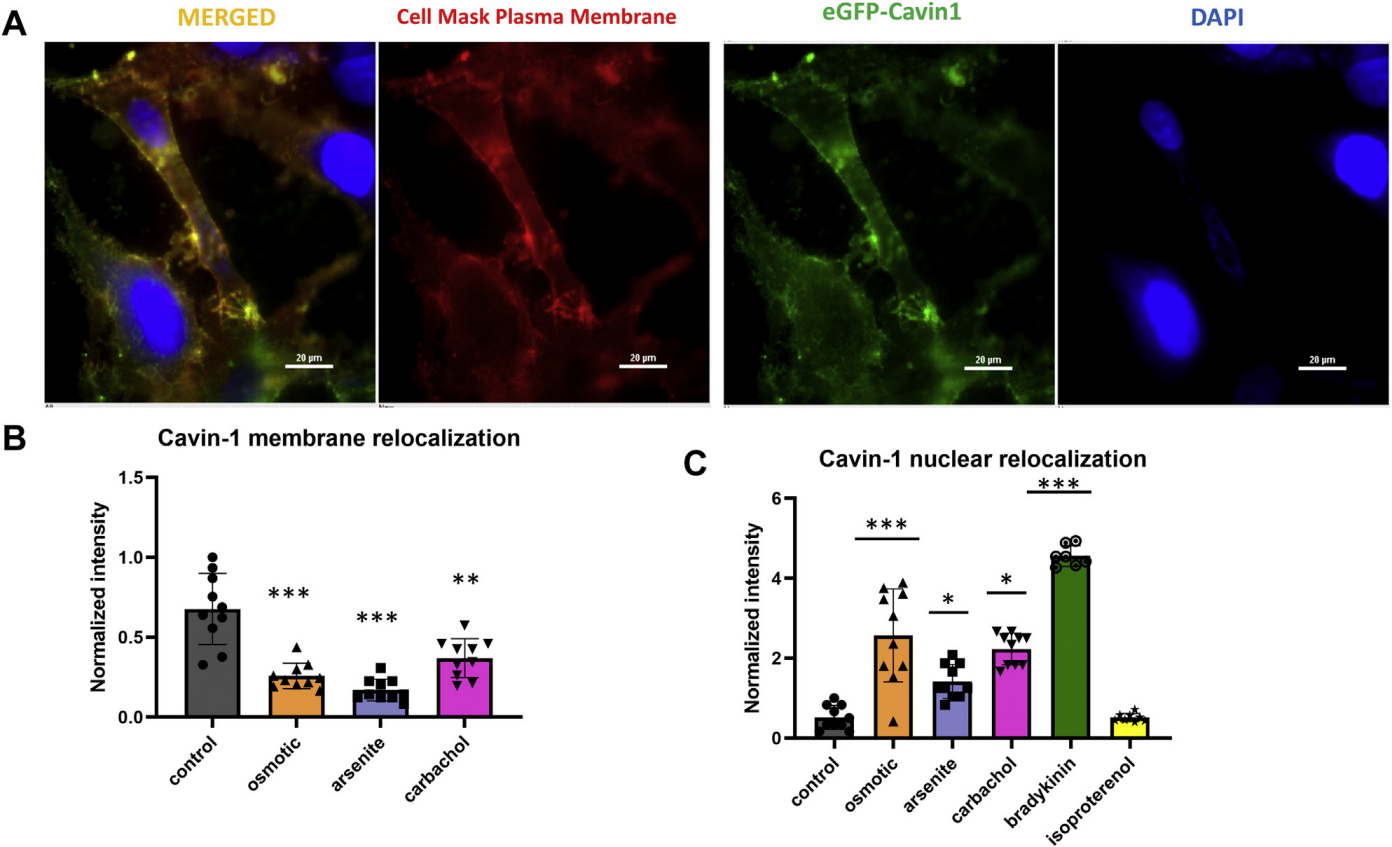

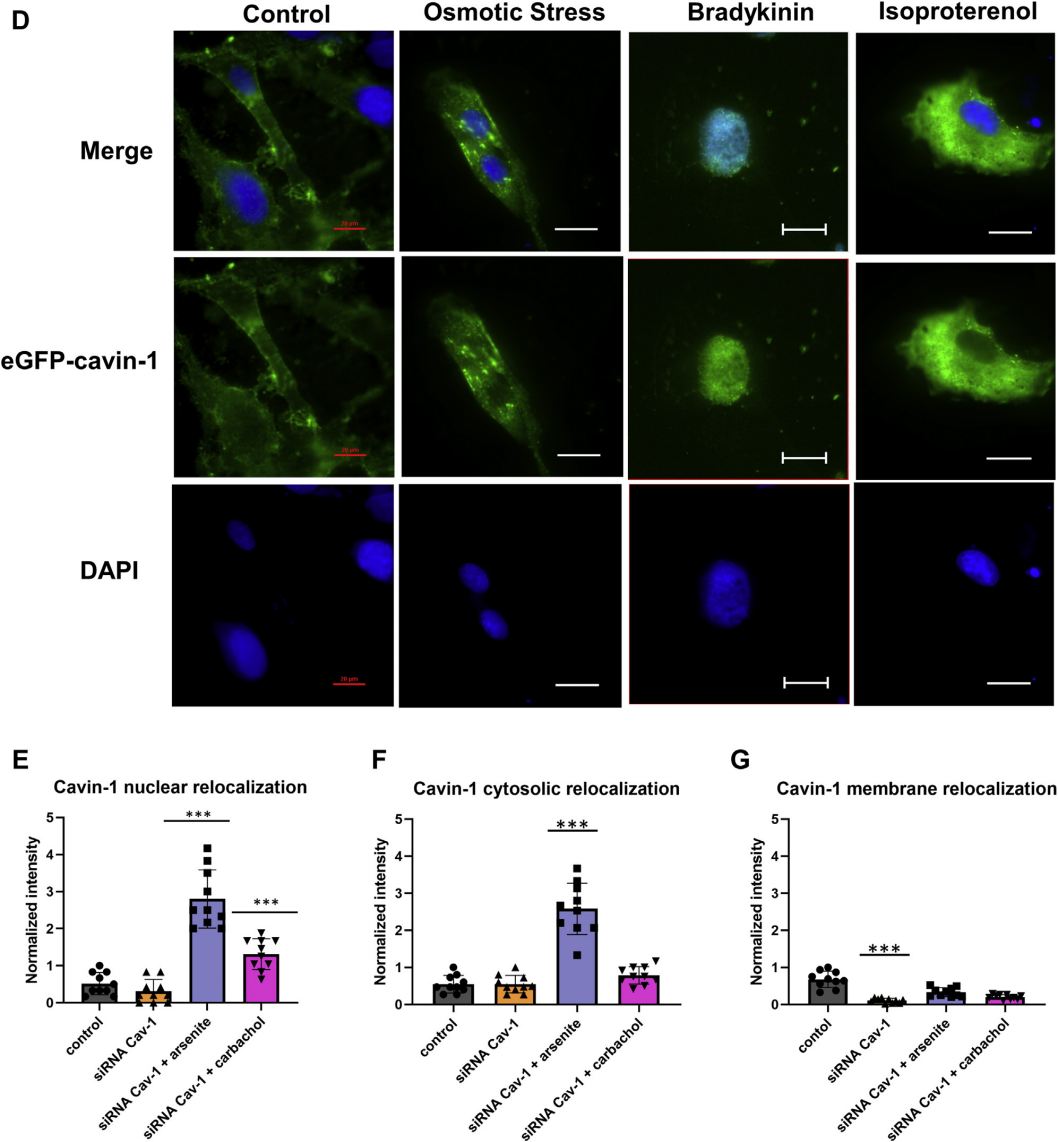
**Cavin-1 relocalization in WKO-3M22 cells.***A*, sample fluorescence images of fixed WKO-3M22 cells transfected with eGFP-Cavin-1 (*green*) and stained with CellMask Deep Red Plasma Membrane stain (*red*) and DAPI (*blue*) under control conditions as obtained at 60X magnification and focusing on the bottom of the cell. Scale bars are 20 μm long. *B* and *C*, cell localization of eGFP-cavin-1 was assessed in cells subjected to hypo-osmotic (*orange*), arsenite (*purple*), Gαq stimulation with carbachol (*pink*), bradykinin stimulation (*green*), and isoproterenol treatment (*yellow*). eGFP-cavin-1 localization was determined by measuring the pixel intensities to coordinates to the plasma membrane, cytosolic, and nucleus compartments based on the location of DAPI and the CellMask plasma membrane stain (see Experimental procedures). Changes in membrane (*B*) and nuclear localization (*C*) in wildtype cells. Intensities were normalized to the plasma membrane control. The *asterisks* correspond to *p* values, with one *asterisk* being 0.1<*p* < 0.5, two *asterisks* meaning *p* < 0.05, and three *asterisks* indicating *p* < 0.001. *D*, sample fluorescence images of fixed WKO-3M22 cells transfected with eGFP-Cavin-1 (*green*) and stained DAPI (*blue*) under control, hypo-osmotic, bradykinin stimulation, and isoproterenol treatment as imaged by epifluorescence with 60X magnification. Note that the control in this figure is the same as the one presented in 2A for ease of comparison. Scale bars are 20 μm long for the control samples and 10 μm long for the rest of the conditions. *E*–*G*, results for eGFP-cavin-1 in transfected cells treated with siRNA(Cav1) where changes in localization in the nucleus (*E*), cytosol (*F*), and membrane compartments (*G*) are shown. Intensities were normalized to the control of the nuclear population. All measurements are an average of three independent experiments that sampled 10 cells, where SD is shown and where the *p* values were determined using a Student *t* test where each dataset was compared to control.

Cavin-1 relocalization studies were carried out in intact Wistar Kyoto smooth muscle cells (WKO-3M22) which are flat and easily imaged. Cells were transfected with eGFP-cavin-1, and shifts in the cellular distribution of fluorescence intensity with hypo-osmotic stress by confocal were measured. Under basal conditions, we find that eGFP-cavin-1 localizes on the plasma membrane with small amounts in the cytoplasm and nucleus (Fig. 2*A*). Because the additional, transfected protein would increase total cellular cavin-1 and may effect localization, we repeated these studies by first downregulating cavin-1 by ∼95% before transfecting with eGFP-cavin-1 (see Fig. S2, *A* and *B*). We then quantified the eGFP-cavin-1 fluorescence intensity on or close to the plasma membrane, the cytosol, and the nucleus (see Experimental procedures). In this case, the observations were similar with the intensity being slightly lower on the plasma membrane than on the nucleus, and no intensity could be detected in the cytosol, suggesting that endogenous cavin-1 is mainly distributed between the plasma membrane and nuclear compartments (see Fig. S2*B*).

When we subjected wildtype eGFP-cavin-1 cells to mild hypo-osmotic stress, a large portion of the plasma membrane GFP-cavin-1 intensity shifts to the cytosol and the nucleus (Fig. 2, *B* and *C*) as seen by tracking the fluorescence intensity relative to the plasma membrane, cytosol, and nucleus (see Experimental procedures). Interestingly, when cells are subjected to bradykinin stimulation, cavin-1 relocalizes to the nucleus. In contrast, when we subjected cells to isoproterenol which does not stimulate Gαq, eGFP-cavin-1 remains outside the nucleus (Fig. 2*D*). To test the idea that cavin-1 shifts to the nucleus when caveola domains are disrupted, we downregulated Cav1 and found that little cavin-1 is associated with the plasma membrane under these conditions (Fig. 2, *E*–*H*). Taken together, these data show that caveola deformation, as well as disassembly, has the potential to promote nuclear localization of cavin-1, allowing it to function as a transcription activator.

We also tested whether other stress conditions impact cavin-1 localization. Because Cav1–Gαq interactions strengthen when activated, we determined whether Gαq stimulation would in turn change cavin-1–Cav1 interactions and cavin-1 localization. To this end, we find that addition of carbachol to activate Gαq promotes relocalization of GFP-cavin-1 to the nucleus (see supporting information) and this relocalization is much more pronounced when Gαq is stimulated with bradykinin, consistent with a high bradykinin receptor in muscle cells (Fig. 2). Note that siRNA(Cav1) does not affect Gαq levels (Fig. S2*D*). Additional cell images are also displayed in Fig. S2*C*. Because both carbachol and bradykinin can stimulate Gαi in addition to Gαq, we stimulated cells with isoproterenol, which does not activate Gαq. In this case, no shift in intensity to the nucleus occurs (Fig. 2, *C* and *D*) although it appears that a population of cavin-1 moves to the cytosol and the underlying mechanism is currently under investigation. We also found that treating cells with arsenite, which promotes a p53 response and is correlated to cavin-1 activity (25), also promotes cavin-1 nuclear localization, but to a lesser extent than Gαq activation. Interestingly, when caveolin-1 is downregulated, cavin-1 nuclear relocalization is more pronounced in arsenite-treated cells than carbachol-stimulated ones (Fig. 2, *E*–*G*).

The data in Figure 2 show that several stress conditions promote movement of cavin-1 from the plasma membrane to the nucleus, which would be expected to increase protein production based on cavin-1’s nuclear function. However, this is not seen in Figure 1 where similar or lower levels of proteins, normalized for actin and changes in total protein content, were observed. Therefore, we investigated other mechanisms through which Cav1 would influence protein levels.

### Transient downregulation of Cav1 or cavin-1 affects the amount of cytosolic RNAs and their size distribution

**Figure 3:**
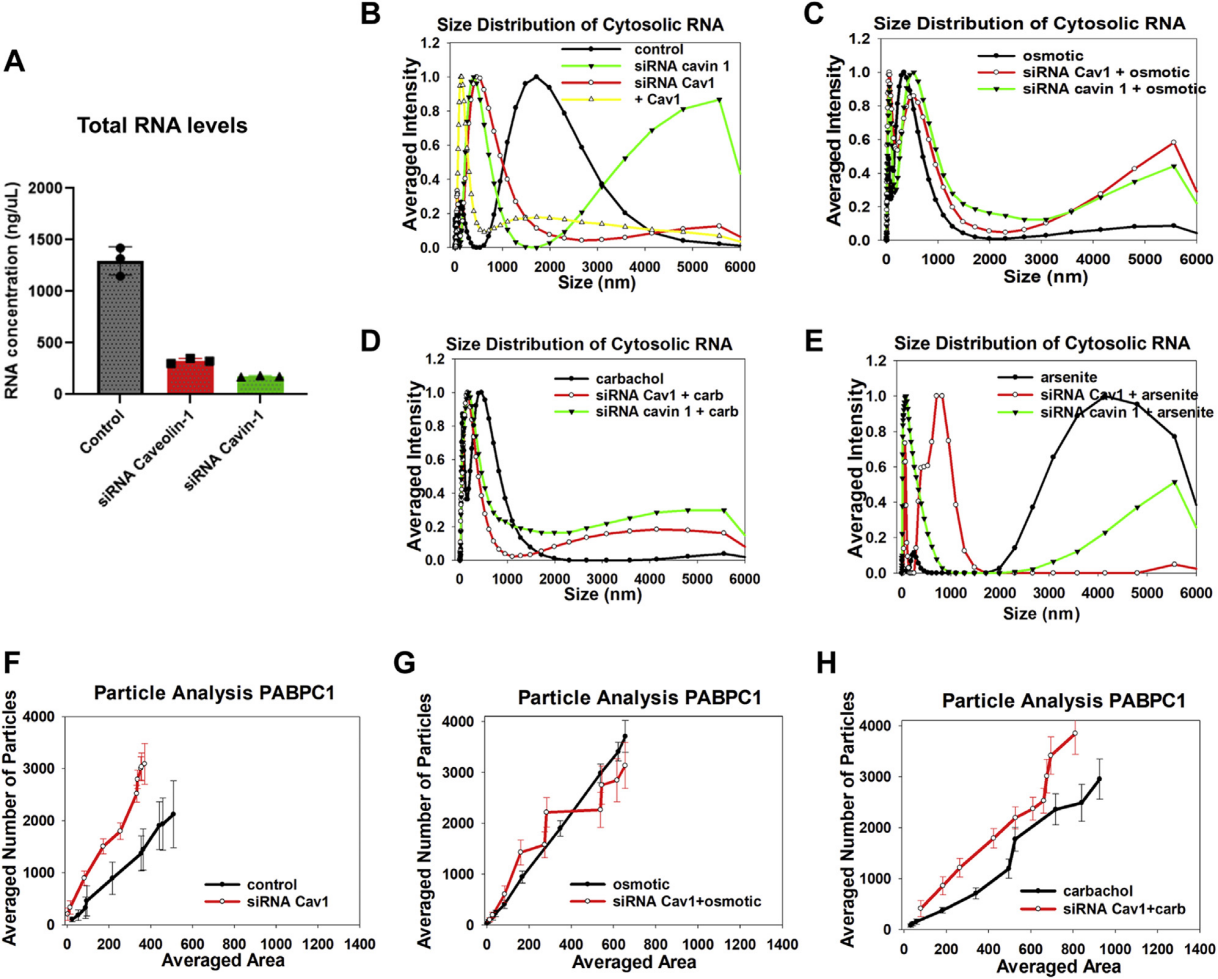
**Cav1 effects cytosolic RNA size distribution and stress granules in WKO-3M22 cells.***A*, RNA from WKO-3M22 cells treated with siRNA (Cav1) (red) and siRNA (cavin-1) (*green*) was extracted and quantified (see Experimental procedures). *B*, normalized DLS spectra showing the size distributions of cytosolic RNA isolated from WKO-3M22 cells under control conditions (*black*), siRNA(Cav1) treated cells (*red*), cavin-1 overexpression (*yellow*), and siRNA(cavin-1) (*green*) downregulation. *C*–*E*, similar study as in (*B*) for cells subjected to hypo-osmotic stress (150 mOsm 5 min), and (*D*) cells subjected to Gαq stimulation with carbachol treatment (5 μM, 10 min) or (*E*) cells subjected to 0.5 mM arsenite. Each sample was scanned 3 times at 10 min per run. The number of independent samples was six per condition. *F*–*H*, the cumulative size and number of particles associated with monoclonal anti-PABPC1 in the cytosol of fixed and immunostained WKO-3M22 cells was measured on a 100x objective and analyzed using Image J (see Experimental procedures). Comparison of cells with siRNA (Cav1) to mock-transfected under basal conditions (*F*), hypo-osmotic stress (150 mOsm, 5 min) (*G*), and stimulation of Gαq by treatment with 5 μM carbachol (*H*). All measurements are an average of three independent experiments that sampled 10 cells, where SD was shown and where the *p* values were determined using ANOVA. DLS, dynamic light scattering.

In the aforementioned studies, we observed that downregulating Cav1 or cavin-1 results in increased cell death by ∼40% and ∼52%, respectively, as estimated by comparing the number of transfected to control cells in 3 to 5 culture dishes. We postulated that this increase in mortality is due to decreased transcription and protein loss caused by reduced levels of cavin-1. To this end, we measured changes in the levels of cytosolic RNA (Fig. 3*A*). These studies were done by isolating the cytosolic fractions of WKO cells, removing the nuclear and lipid components, and isolating the RNA after protein digestion and lipid solubilization (see Experimental procedures). We find that downregulating Cav1 by 60% and cavin-1 by 38%, as estimated by Western blotting in WKO-3M22 cells (Fig. S2*A*), resulted in a 5-fold reduction of cytosolic RNA, while downregulating cavin-1 by 94% and Cav1 by 74% resulted in a ∼13-fold reduction (Fig. 3*A*). Notably, subjecting cells to osmotic stress also reduced cytosolic RNA levels even though more cavin-1 was nuclear (Fig. 2*C*).

We characterized relative size distributions of the cytosolic RNA populations by dynamic light scattering (DLS). We find that the transcripts effected by Cav1 downregulation are smaller in size, suggesting a higher level of processing. The RNA seen when cavin-1 is downregulated is very large, suggesting reduced processing, and the relative amount of the larger-sized population decreases with the application of stress (Fig. 3, *B*–*E*). The loss in RNA with Cav1/cavin-1 is consistent with reduced proliferation due to reduced transcription, and the shifts in the sizes of cytosolic RNA suggest that Cav1/cavin-1 levels impact RNA processing. Additionally, transfection of cavin-1 to WKO cells treated with siRNA(cavin-1) showed DLS spectra similar to control (Fig. S2*F*). We note that the percentage of rRNA to cytosolic RNA levels is reduced when Cav1 or cavin-1 is downregulated as indicated by weaker contribution of the 18S and 28S ribosomal RNA bands (Fig. S2, *E* and *F*).

### Transient loss of Cav1/cavin-1 impacts stress responses

The changes in size distributions in Figure 3 suggest that Cav1/cavin-1 levels or stress conditions impact cytosolic RNA processing. To better understand these changes, we looked at the two major mechanisms used by cells to handle cytosolic RNA: processing bodies (p-bodies) and stress granules. P-bodies are associated with RNA degradation as well as RNA storage (26), and stress granules are halted ribosomal RNA complexes that protect mRNA during stress conditions, such as the hypo-osmotic conditions used here (27). Stress granules and p-bodies have an overlapping storage function, and both contain Ago2 (28) which either stalls mRNA translation or degrades mRNA depending on the amount of base pairing between the Ago2-bound miR and the mRNA. Since both stress granules and p-bodies require RNA, their assembly will be inhibited by the reduced RNA levels accompanying cavin-1 downregulation.

We first tested whether Cav1/cavin-1 downregulation changes the number and size of stress granules under basal conditions. These studies were carried out by immunostaining WKO-3M22 cells treated with either control or Cav1 siRNA and immunostaining for the stress granule marker, polyadenylate-binding protein (cytosolic)-1 (PABPC1). The size and area of PABPC1 particles were then quantified from high-resolution fluorescence confocal images (see Experimental procedures). In Figure 3*F*, we show a comparison of control and siRNA(Cav1) in cells under basal conditions. We find that loss of Cav1/cavin-1 results in more numerous but smaller particles consistent with smaller RNAs seen by DLS.

We then determined whether reducing Cav1/cavin-1 levels change the ability of cells to form PABPC1 particles (*i.e.*, stress granules) under hypo-osmotic stress or carbachol stimulation (Fig. 3, *G* and *H*). We find that reduced Cav1/cavin-1 levels did not affect particle formation in cells subjected to osmotic stress and produced a small increase in the number of fewer particles in cells stimulated with carbachol. These results suggest that the reduced levels of GAPDH and Hsp90 caused by Cav1 downregulation (Fig. 1) are not due to confinement of their mRNAs into large stress granules.

The reduction in cytosolic RNA may result in smaller stress granules that might be missed in immunostaining studies. In a second series of studies, we monitored stress granule formation in live cells using the stress granule assembly protein G3BP1 tagged with eGFP. This construct allowed us to monitor small aggregates by a fluorescence correlation method (number and brightness [N&B]) which senses small aggregates. N&B is a fluorescence method which follows the diffusion of a fluorophore in an image series and compares the number of photons associated with this diffusion to a monomeric control, such as free eGFP (29). This method allows us to determine small particles that might be missed in confocal imaging.

**Figure 4:**
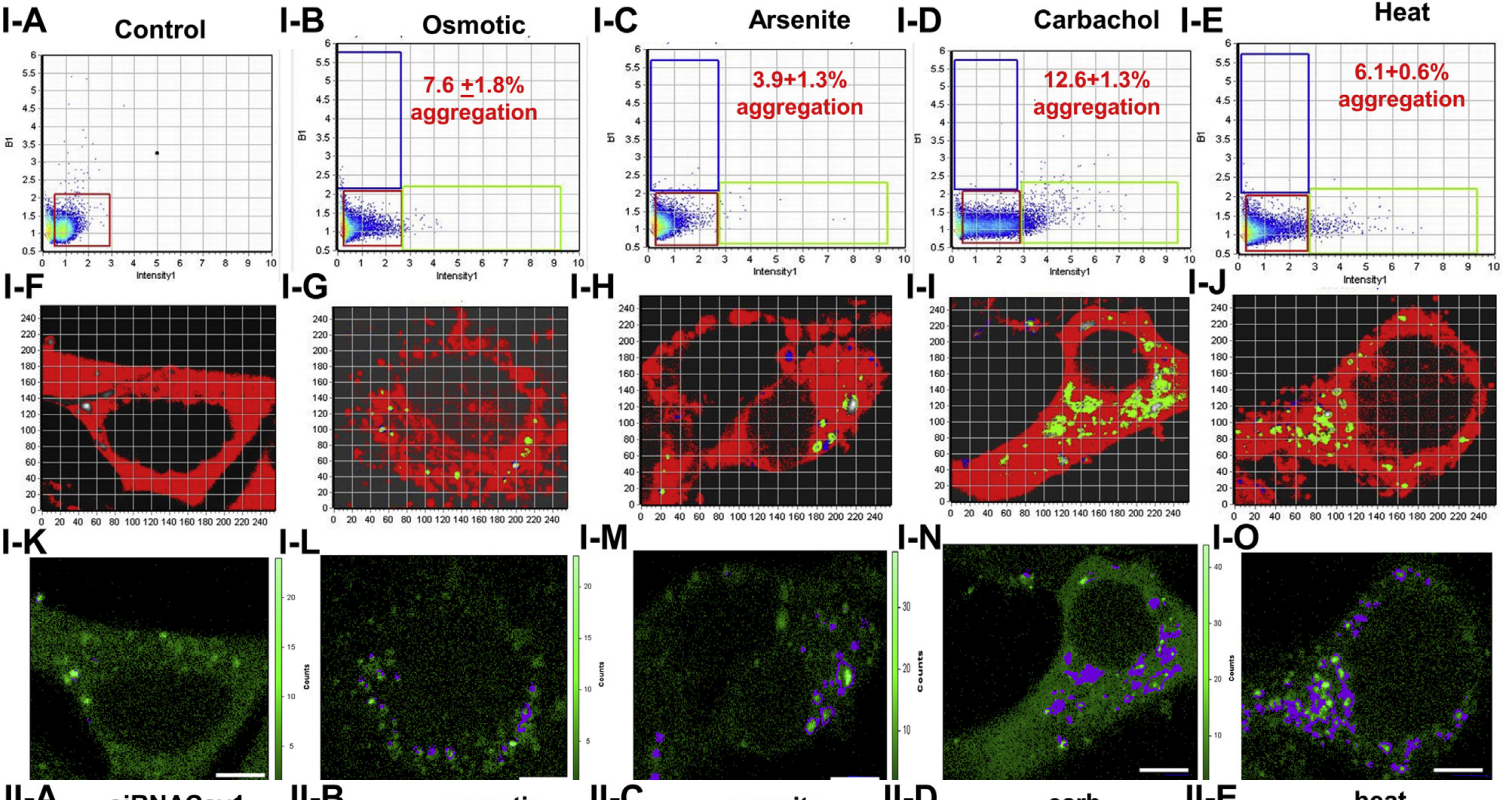

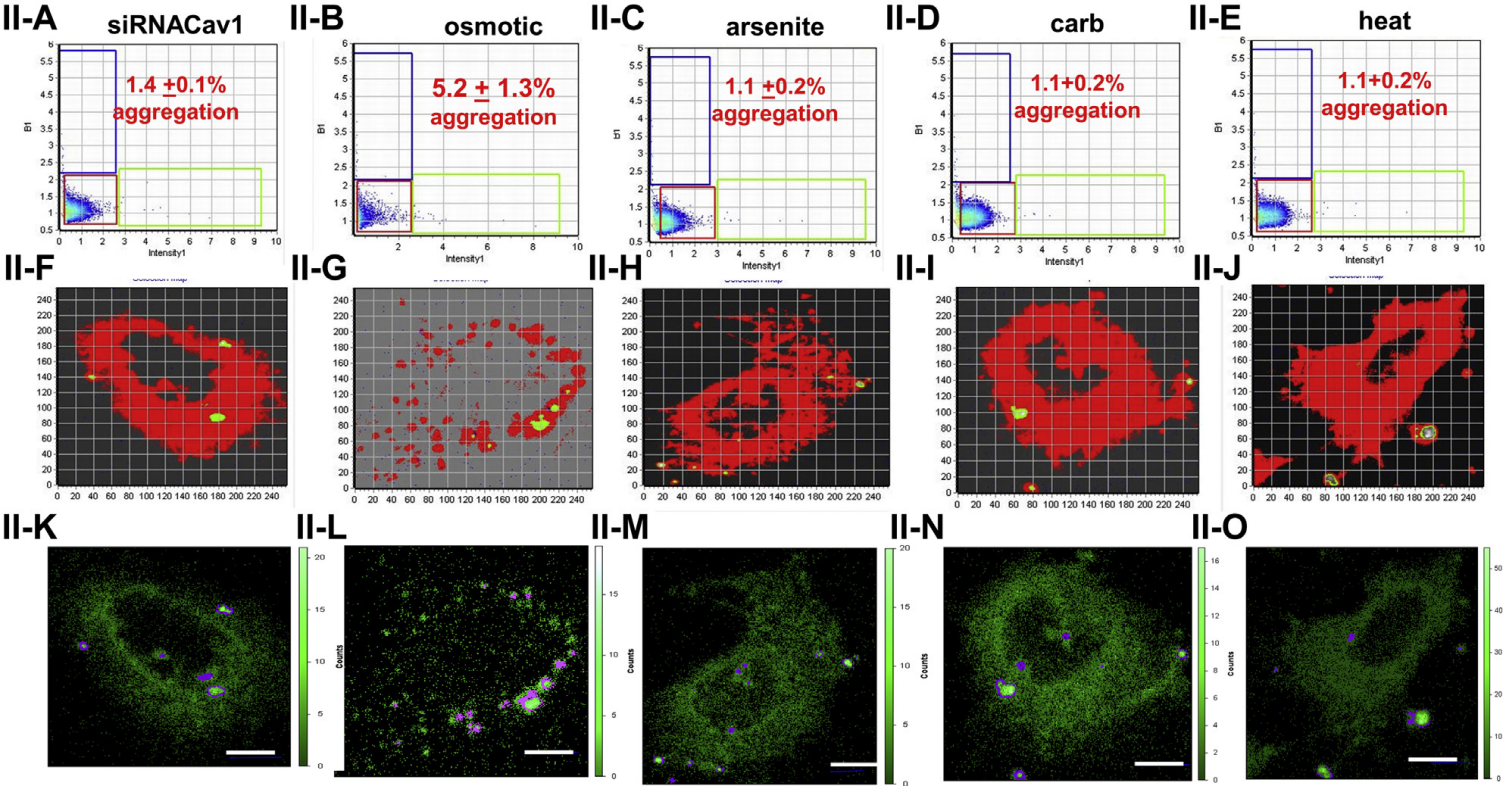
**N&B analysis of eGFP-G3BP1 aggregation in WKO-3M22 cells.** Part I: *A*–*J*, the *top panels* (I-A –I-E) show graphs of the brightness *versus* intensity with the pixels of the *colored boxes* corresponding to the specific regions in the cells (I-F – I-J) using SIM-FCS 4 software. *K*–*O*, the *bottom panels* show the corresponding fluorescence microscopy images in ISS (I-K –I-O). The *red box* corresponds to monomeric eGFP-G3BP1, while points outside this box and in the *green* and *blue boxes* correspond to higher order species. Panels I-A, I-F, and I-K are control cells (n = 8); panels I-B, I-G, and I-L are cells subjected to hypo-osmotic stress (150 mOsm, 5 min) (n = 6); panels I-C, I-H, and I-M are cells subjected to arsenite stress (0.5 mM, 10 min) (n = 6); panels I-D, I-I, and I-N are cells subjected to carbachol stimulation (5 μm, 10 min)(n = 6); panels I-E, I-J, and I-O are cells subjected to heat shock (42 °C, 60 min)(n = 6); scale bars are 10 μm long. Part II is similar to Part I for cells treated with siRNA(Cav1) (n = 6). Scale bars are 10 μm long. N&B, number and brightness.

In Figure 4, we show control cells transfected with eGFP-G3BP1 and subjected to various stress conditions. The amount of aggregation with each stress condition correlates to amount of cavin-1 relocalization seen in Figure 2; carbachol stimulation showed the highest amount of aggregation, followed by osmotic stress and then arsenite. When Cav1/cavin-1 was downregulated, no changes in G3BP1 aggregation were observed except for osmotic stress which showed a small, residual change that might be due to residual Cav1. These results suggest that lowering Cav1/cavin-1 levels make cells less able to adopt mechanisms that protect them from environmental stress.

**Figure 5:**
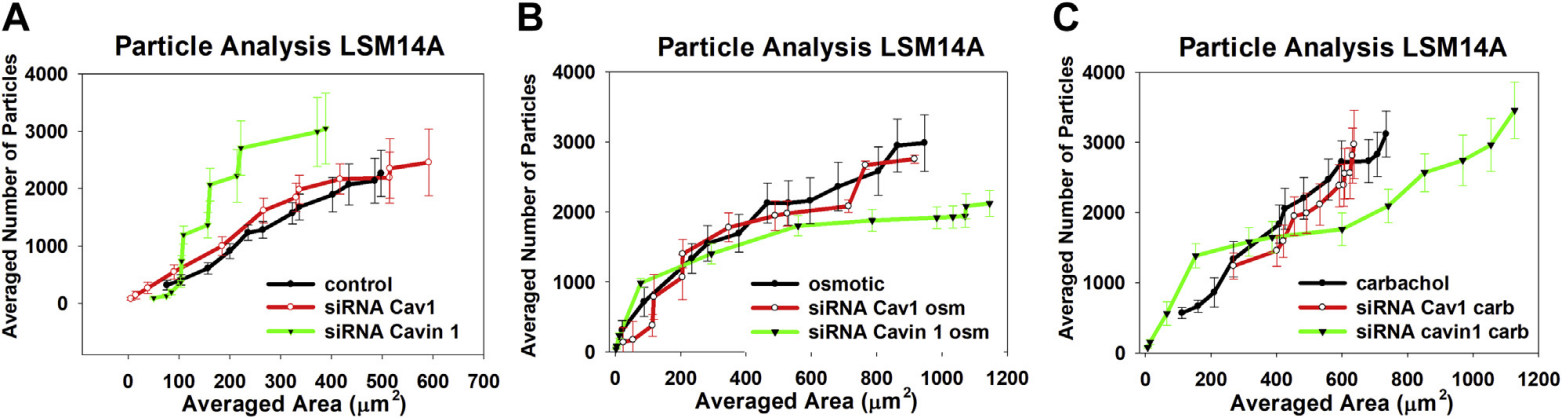
**Cavin-1 impacts LSM14A containing P-body formation**. The size and number of particles associated with monoclonal anti-LSM14A in the cytosol of fixed and immunostained WKO-3M22 cells was measured on a 100x objective and analyzed using ImageJ (see Experimental procedures). *A*–*C*, comparison of mock treated and cells treated with siRNA(cavin-1) or siRNA(Cav1) under basal conditions (*A*), hypo-osmotic stress (150 mOsm, 5 min) (*B*), and stimulation with 5 μM carbachol (*C*). All measurements are an average of three independent experiments that sampled 10 cells, where SD was shown and where the *p* values were determined using ANOVA.

It is also possible that reduced cytosolic RNA with Cav1/cavin-1 knockdown will impact p-bodies. We used the p-body marker, LSM14A, which is associated with inactive mRNA storage, to monitor the formation of p-bodies on the micron scale (30, 31). High-resolution confocal images were analyzed to determine the number and area of LSM14A particles. Compared to control cells, downregulating Cav1 had little effect on p-bodies, but downregulating cavin-1 results in a greater number of LSM14A particles (Fig. 5), showing that the available RNA is not being processed, which is consistent with reduced ribosome activity (see Discussion). Additionally, we find that carbachol stimulation increases formation of LSM14A containing p-bodies, while osmotic stress has no effect.

The aforementioned studies indicate that Cav1/cavin-1 levels primarily affect the formation of stress granules that form under various environmental conditions rather than p-bodies. Therefore, we sought to investigate the effect of Cav1/cavin-1 on the formation of large and small aggregates containing Ago2, which aggregates in cells subjected to osmotic stress or Gαq activation (32). We monitored stress-induced aggregation of eGFP-Ago2 in live cells with Cav1/cavin-1 downregulation. When viewing Ago2 aggregates in control cells by fluorescence microscopy, we find that environmental stress does not significantly impact the size and number of Ago2 particles (Table 1 and Fig. S3) and only minor changes, similar to PABPC1 particles, are seen for cells treated with siRNA (Cav1). When we view small Ago2 particles in control cells by N&B analysis, we find large effects due to osmotic and carbachol stress but little change with heat and arsenite (Table 1). These results show that downregulation of Cav1/cavin-1 reduces stress-induced aggregation, most likely due to reduced cytosolic RNA levels (Fig. S4).

**Table 1:** Stress responses quantified by particle analysis and N&B

Stress condition	Particle analysis		N&B	
	Ago2	PABPC1	Ago2	G3BP1
Control	1.8	4.2	n/a	n/a
Osmotic	1.6	**5.6**	**34.0**	**7.6**
Carbachol	1.7	**3.2**	**19.0**	**12.6**
Arsenite	1.5	2.9	5.0	3.9
Heat	1.5	3.1	3.9	**6.1**
siRNA Cav1	2.5	**7.6**	5.3	1.4
siRNA Cav1 + osmotic	1.7	4.3	2.3	**5.2**
siRNA Cav1 + carbachol	3.1	4.4	0.4	1.1
siRNA Cav1 + arsenite	1.7	**6.0**	1.3	1.1
SiRNA Cav1 + heat	**5.3**	4.5	**16.4**	1.1
siRNA cavin-1	2.3	3.4	**9.9**	1.3
siRNA cavin-1 + osmotic	1.4	2.9	3.6	1.6
siRNA cavin-1 + carbachol	**7.3**	**7.4**	4.0	1.2
siRNA cavin-1 + arsenite	1.8	**5.9**	2.9	1.2
siRNA cavin-1 + heat	3.0	3.0	2.6	1.2

Shown are values for the slopes of the particle analysis curves (*i.e.*, the number *versus* area of particles) and the N&B values. Changes over 4-fold are in bold.

### Cavin-1 KO cells show widespread phenotypic changes as well as adaptive behavior

We obtained a strain of mouse embryonic fibroblasts (MEFs) where cavin-1 was knocked out using CRISPR/Cas9 genome editing technology (19). These KO cells have a 3-fold longer doubling time and a more trapezoid morphology than their wildtype counterparts where the circularity dropped from 0.78 + 0.2, n = 16 to 0.55 + 0.3, n = 16. Additionally, we were unable to visualize caveolae by Cav1 immunostaining which is consistent with the absence of these caveolae, and these cells had a 55%-68% lower level of Cav1 as quantified by Western blot (Fig. S2*F*). Surprisingly, we find that the cytosolic RNA levels of the KO cells were identical to the wildtype in sharp contrast to transient downregulation of Cav1/cavin-1.

### Cavin-1 KO cells show different cytosolic RNA size distributions

**Figure 6:**
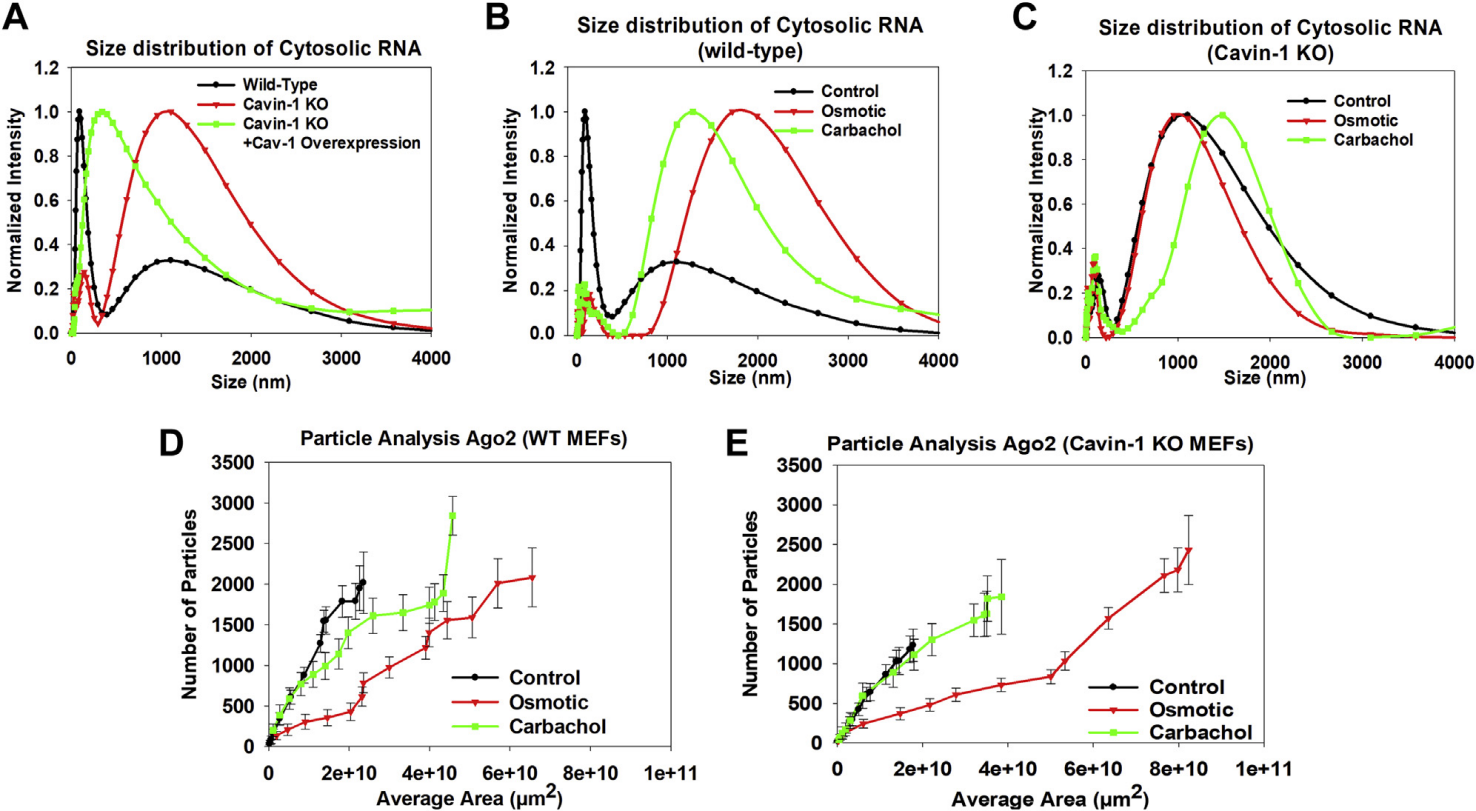
**RNA size distribution and formation of eGFP-Ago2 particles in wildtype and cavin-1 KO MEFs.***A*, normalized DLS spectra showing changes in the size distribution of cytosolic RNA isolated from MEFs for mock-treated control cells (*black*), cavin-1 knockout cells (*red*), and cavin-1 KO cells overexpressing Cav1 (*green*). *B*, DLS spectra of cytosolic RNA from wildtype MEFs under control conditions (*black*), hypo-osmotic stress (150 mOsm 5 min, red), cells overexpressing of constitutively active Gαq (*green*). *C*, an identical study showing the size distribution of cavin-1 KO cells under control conditions (*black*), hypo-osmotic stress (150 mOsm 5 min) (*red*), and carbachol stimulation (5 μM for 10 min, *green*). Each sample was scanned 3 times at 10 min per run. The number of independent samples was six per condition. *D*, cumulative size and number of particles associated with anti-Ago2 in the cytosol of MEFs were measured on a 100x objective and analyzed using ImageJ (see Experimental procedures) showing treatment of wildtype cells with hypo-osmotic stress (150 mOsm, 5 minutes, *red*) and Gαq stimulation (5 μM carbachol, 10 min, *green*). *E*, treatment of cavin-1 KO cells to 5 min of osmotic stress (150 mOsm) and carbachol stimulation (5 μM carbachol, 10 min, *green*). Measurements are an average of three independent experiments that sampled 10 cells. *p* values were determined using ANOVA. DLS, dynamic light scattering; MEF, mouse embryonic fibroblast.

The adaptive changes in cytosolic RNA in the KO cells gave us the opportunity to understand the effects of cavin-1 at constant levels of cytosolic RNA. We find that even though the cytosolic RNA levels in the cavin-1 KO are the same as wildtype, their size distributions are very different (Fig. 6, *A*–*C*). Cytosolic RNA of wildtype cells has two populations: a major one at small RNA sizes and a minor one at larger sizes. These populations are reversed in the KO cells; the peak at small RNA sizes was only one-third as high as the one at larger sizes. When we increase the expression of cavin-1 by overexpressing Cav1 in the KO cells, we find a shift in RNA sizes toward wildtype values.

The shift to larger cytosolic RNAs sizes in the KO cells could be due to reduced processing. To test this idea, we subjected wildtype cells to carbachol or hypo-osmotic stress to halt mRNA processing. These stresses reduced the amount of small RNAs consistent with the halting of Ago2 activity due to stress granule formation. The loss of small cytosolic RNAs in the wildtype cells results in a DLS spectrum similar to unstressed KO cells. In contrast, subjecting cavin-1 KO cells to stress did not significantly change the cytosolic RNA size distribution. Thus, we interpret the higher sizes in the KO cells as being caused by reduced RNA processing (Fig. 6, *D* and *E*).

The large sizes of cytosolic RNA may be consistent with stalled ribosomal complexes (*i.e.*, stress granules), and we determined the ability of the cavin-1 KO cells to form stress granules. Particle analysis of wildtype and KO cells showed Ago2 particles are similar in size and number in cells under basal conditions and cells subjected to hypo-osmotic while the KO cells produced fewer particles under carbachol stimulation than wildtype cells (Table 1 and Fig. S5). This reduction is consistent with the reduced levels of Gαq activation due to the loss of caveolae. The size of Ago2 particles formed was greatly enhanced upon Cav1/cavin-1 upregulation, indicating some type of a compensatory mechanism (Table S1 and Fig. 5). These cells were also highly sensitive to arsenite, suggesting that one of the adaptive mechanisms used by these cells is energy dependent. We also viewed small eGFP-Ago2 particles in wildtype and cavin-1 KO cells by N&B (Table S1). As compared to control cells, we find that the cavin-1 KO cells have eGFP-Ago2 particles that contain more fluorophores and are more intense. When cells are subjected to hypo-osmotic stress, the KO cells show enhanced aggregation which was slightly reversed with Cav1 overexpression (Fig. S6). Thus, even though cavin-1–depleted cells have less particles on the micron scale, they have a larger number of smaller particles, and environmental stress promotes the formation of small Ago2 particles.

## Discussion

In this work, we have begun to delineate the complex connections between caveolae, mechanical stretch, and cell physiology using hypo-osmotic stress to deform caveolae. Caveolae provide mechanical strength to cells and serve as platforms that localize signaling proteins (1). Several years ago, we found a connection between these two functions when we observed that Cav1/3 enhances Gαq/calcium signals and that deformation of caveolae disrupts Cav–Gαq interactions (9). Here, we show that caveolae have an additional role by indirectly helping cells sense and respond to environmental stress conditions by modulating translational and transcriptional processes.

These studies were initiated by work showing that elevating cytosolic PLCβ reverses RNA-induced silencing of GAPDH but not Hsp90 through its inhibition of a promotor of RNA-silencing, C3PO (12). Activation of Gαq drives PLCβ to the plasma membrane promoting both RNA-induced silencing and stress granule formation (12, 33). Because Cav1/3 stabilizes activated Gαq (5), we postulated that its presence might also affect protein production by modulating Gαq activity. Our initial studies measured the effect of reduced Cav1 on the expression of two housekeeping proteins GAPDH and Hsp90 and the growth-associated protein Ras. As detailed below, the impact of Cav1 on the basal levels of these proteins was difficult to quantify because of its broad effect on cellular RNA, and so we focused on changes in the levels of these proteins when caveolae were deformed under mild osmotic stress. By comparing to control cells, we find that levels of all three proteins are influenced by Cav1 downregulation. The impact of Cav1 on GAPDH and Hsp90 does not appear to primarily involve the Gαq/PLCβ signaling pathway since cells where either Gαq or PLCβ1 were downregulated showed either minor or no changes as compared to controls, although our studies suggest that Ras levels might be influenced by both Gαq and Cav1. Thus, these initial studies suggest that Cav1 may act through pathways distinct from Gαq/PLCβ to impact the levels of specific cellular proteins.

We searched for mechanisms that connect caveolae to protein levels in another cultured smooth muscle cell line that are easily imaged. We noted that cavin-1 plays a dual role in cells by stabilizing caveola domains on the plasma membrane and by promoting the transcription of ribosomal RNA in the nucleus (3, 19). Because expression of Cav1 and cavin-1 are linked, we were not surprised to find that downregulating Cav1 results in a dramatic decrease in cytosolic RNA levels, which may influence cellular levels of proteins like GAPDH, while downregulating cavin-1 causes a much larger reduction of RNA. The ability of cavin-1 to regulate transcription allowed us to use the level and properties of cytosolic RNA as a readout for nuclear cavin-1 activity.

Cavin-1 has been found to be released from the plasma membrane when caveola domains disassemble or upon phosphorylation during insulin signaling (19). Here, we worked at osmotic conditions where caveolae deform enough to disrupt Cav1–Gαq interactions and focused on cavin-1 relocalization in real time in intact cells (9). We find that even these mild conditions promoted release of cavin-1 from the plasma membrane to the cytosol and nucleus. Not only did osmotic stress allow for cavin-1 relocalization, but carbachol stimulation did as well suggesting that strengthening Cav1–Gαq interactions weakened contacts with cavin-1. As expected, activation by isoproterenol did not promote nuclear localization of eGFP-cavin-1. We were surprised to find that arsenite treatment, which has pervasive effects in cells, also promotes cavin-1 relocalization to the nucleus. This relocalization may be due to initiation of cellular mechanisms to alleviate cell toxicity or due to disintegration of caveola–cytoskeletal contacts (34, 35, 36).

While cavin-1’s functions on the plasma membrane and in the nucleus are known, and the large population of this protein localizes in these two compartments, its role in the cytosol has not yet been determined. It is possible that the cytosolic population acts as a reservoir for the other cellular compartments. It is notable that cavin-1 has been found to form complexes with cavin-2 in the cytosol (37). More recent work found that cavin-3, which interacts with both cavin-1 and caveolin-1 that can also be released from the plasma membrane upon caveola disassembly, interacts with a large number of cytosolic proteins and some of these interactions may overlap with cavin-1 (38, 39). Pertinent for this study is the argument that cytosolic cavin-1 does not appear to directly mediate stress granule formation since we have not detected cavin-1 in protein complexes associated with either PLCβ1 or Ago2 (32).

Our studies showed that reducing Cav1/cavin-1 levels and subjecting cells to osmotic stress reduced GAPDH and Hsp90 levels relative to controls but increased Ras. We do not believe that these differential effects on these transcripts are specific to cavin-1 but instead are indirect through other pathways, or due to the rate of translation of transcript caused by reduced ribosomes levels. We explored this idea by assessing the relative size distribution of cytosolic RNAs. In WKO cells, lowering cavin-1 promotes the appearance of very large RNAs, while overexpressing cavin-1 increases small RNAs due to enhanced processing consistent with enhanced ribosomal activity. Reducing cytosolic PLCβ through stimulation of Gαq or application of osmotic stress shows the same shift toward smaller RNAs due to increased RISC activity, while halting processing by arsenite causes a shift to very large sizes. These shifts are consistent with cavin-1’s role in promoting cytosolic RNA processing by increasing ribosomal RNA levels (18).

Cavin-1 is highly expressed in proliferative tissues and ones rich in caveolae such as prostate cancer cells, rhabdomyosarcoma, endothelial cells, etc. (40, 41, 42, 43). Aside from making cells more susceptible to damage from mechanical stretch, downregulating cavin-1 would be expected to slow cell growth due to reduced ribosomal RNA output, and these characteristics were observed in the MEF cavin-1 KO cells (19). Even though they grew much slower than wildtype, the KO MEFs had similar levels of cytosolic RNA, showing that these cells have adapted to reduced ribosomal RNA transcription. We noted that the sizes of the RNAs in the cavin-1 KO MEFs were much larger than control, suggesting a reduction in processing. When we overexpress Cav1 in the KO MEFs, we find a shift in RNA sizes toward control cells. This shift is not due to increased expression of cavin-1 by Cav1 overexpression because the cavin-1 gene has been eliminated from these cells. One possibility is that Cav1’s stabilization of Gαq shifts the PLCβ population to the plasma membrane promoting RISC activity (12), and this idea is supported by previous FRET measurements (32).

Stress granule formation is initiated by cytosolic mRNA, and so it was not surprising that reducing Cav1/cavin-1 results in cells that have impaired stress responses. We tested the importance of Cav1/cavin-1 on micron-sized particles by imaging marker proteins, and for MEFs, we were also able to image smaller aggregates by N&B analysis of fluorescent-tagged markers. Under basal conditions, the cavin-1 KO MEFs showed a greater number of small aggregates of Ago2 but a lower number of larger aggregates than wildtype cells and transfecting with Cav1 did not affect the results since cavin-1 is required for caveolae.

**Figure 7:**
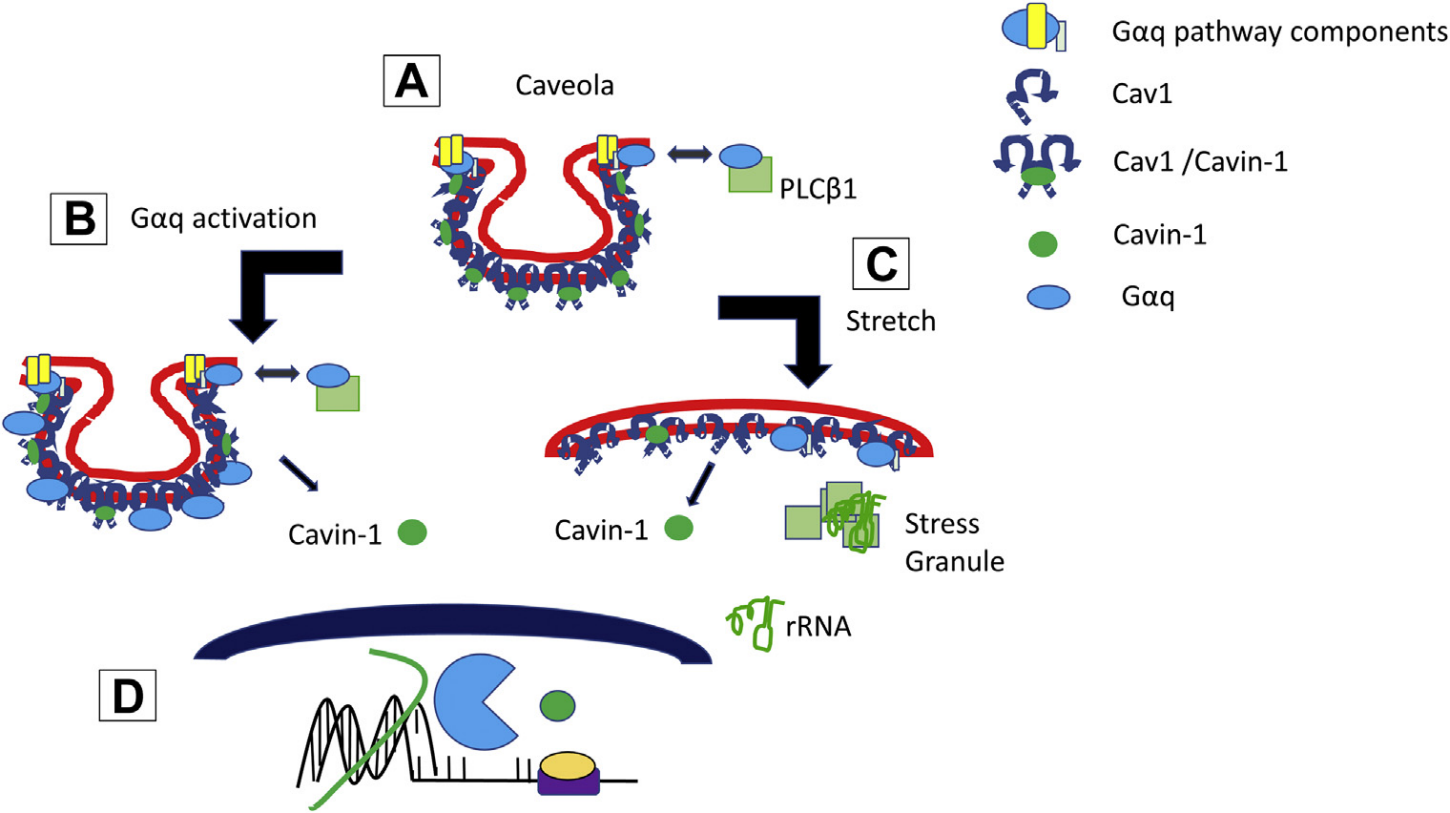
**Model of cavin-1 relocalization with stretch.***A*, Gαq and PLCβ localize in caveolae. *B*, Gαq activation allows the transferring of activity to cavin-1 causing relocalization from the plasma membrane to the nucleus. *C*, mechanical stretch flattens the domains releasing cavin-1 from the plasma membrane and shifting PLCβ to the cytosol which leads to a reduced number of stress granules. *D*, cavin-1 relocalizes to the nucleus to influence transcription of specific genes that lead to changes in cell structure.

The overall goal of this study was to understand how caveola transmits mechanical stress into cells and their ability to mediate Gαq signals. We used a mild osmotic stress where caveolae are deformed but not completely disassembled and one that emulates physiological conditions that can occur in the epithelial cells in the digestive tract and kidney and also in fish and invertebrates (9, 44). While we knew that this stress would reduce Gαq/calcium signals, we were surprised to find that caveolae changed the ability of cells to handle stress as seen by changes in protein content, changes in cytosolic RNA, and changes in stress granule number and area. Our data indicate that these changes are mediated by two factors: relocalization of cavin-1 from the plasma membrane to the nucleus to allow increased rRNA production and increased translation and increased RNA levels in the cytosol as described in the model shown in Figure 7. The nature of the specific proteins translated is unclear and may depend on competing transcripts, miRs, and the rate of translation. Low levels of rRNA reduce the formation of ribosomes allowing cytosolic mRNA to be susceptible to inclusion in p-bodies or degradation by RISC (45). The rRNA resulting from nuclear cavin-1 provides a protective effect for cells by increasing cytosolic RNA levels allow for stress granule formation, although reduction in ribosomes may shift more mRNA into p-bodies as our studies indicate. It is notable that caveolae are prevalent in cells where expression of Gαq is high. Thus, normal Gαq activation by agents such as bradykinin, angiotensin II, histamine, etc. (46), and the studies here suggest that normal, Gαq activation helps cycle cavin-1 to the nucleus in response to these signals. Taken together, these studies show that cavin-1 can act as a sensor that communicates environmental stress to the nucleus that allows cells to better initiate stress responses.

## Experimental procedures

### Cell culture

Rat aortic smooth muscle (A10) cells were purchased from ATCC and used as described (8). Wystar Kyoto rat 3M22 (WKO-3M22) cells, originally obtained from ATCC, were a generous gift from Dr Marsh Rolle. MEFs were a generous gift from Dr Liu Libin (Boston University School of Medicine). WKO-3M22 cell lines were cultured in high-glucose DMEM (Corning) without L-glutamine with 10% fetal bovine serum (FBS), 1% sodium pyruvate, 1% nonessential amino acids (VWR), and 1% L-glutamine (VWR). MEFs were cultured in high-glucose DMEM (Corning) without L-glutamine with 10% FBS, 1% sodium pyruvate, and 1% penicillin–streptomycin. All cells were incubated at 37 °C in 5% CO_2_.

### Plasmids and stains

EGFP-human-Argonaute-2 (eGFP-Ago2) was purchased from (Addgene plasmid # 21981) and was prepared in the laboratory of Philip Sharp (MIT). MCherry-Ago2 was a gift from Alissa Weaver (Vanderbilt University). EGFP-G3BP1 was purchased from Addgene (plasmid #119950) and was prepared in the laboratory of Jeffrey Chao (Friedrich Miescher Institute). EGFP-cavin-1 was a generous gift of Dr Liu Libin (Boston University School of Medicine). CellMask Deep Red Plasma Membrane Stain was purchased by Thermofischer (cat# C10046) for the localization experiments. Plasmid transfections and siRNA knockdowns were done using Lipofectamine 3000 (Invitrogen) in antibiotic-free media. Medium was changed to one containing antibiotic (1% penicillin/streptomycin) 12 to 18 h post transfection.

### Application of stress conditions

For the control measurements, the medium was replaced with Hank’s Balanced Salt Solution (HBSS) before imaging. For hypo-osmotic stress conditions, the medium was diluted with 50% water for 5 min before it was removed and replaced with HBSS for imaging. For arsenite treatment, a stock solution of 100 mM arsenite in water was prepared and a total of 10 μl of stock solution was added to 2 ml of media. Cells were exposed to a final concentration of 0.5 mM arsenite for 10 min before the medium was removed and replaced by HBSS for imaging. For Gαq stimulation, 10 μl of a stock solution of 1 mM carbachol was added to 2 ml of media for a final concentration of 5 μM. Bradykinin was also used to stimulate Gαq by adding 1 μl of a stock solution of 10 mM bradykinin to 2 ml of media for a final concentration of 5 μM. For isoproterenol treatment, 10 μl of a stock solution of 1 mM carbachol was added to 2 ml of media for a final concentration of 5 μM. For the heat shock, cells were incubated at 40 °C for 1 h.

### RNA extraction and DLS

DLS measurements were carried out on a Malvern Panalytical Zetasizer Nano ZS instrument. For these experiments, total RNA from WKO-3M22 cells was extracted following the instructions from the Qiagen Mini Kit (Cat #: 74104). Prior to RNA extraction, cells were exposed to stress conditions. For these measurements, approximately 50 μL of extracted RNA in RNase free water was added in a Hellma Fluorescence Quartz Cuvette (QS-3.00 mm). Each sample was run 3 times, 10 min per run.

### Quantification of cavin-1 cellular localization by fluorescence imaging

Cell localization of eGFP-cavin-1 was assessed by combining multiple images of cells whose compartments were identified with fluorescent markers to the nucleus (DAPI) and the plasma membrane (CellMask) and tracking the shifts in eGFP-cavin-1 fluorescence intensity distribution relative to these coordinates in real time when cells are subjected to various stress conditions. A horizontal line profile (H-Line Profile) was generated with counts *versus* pixel X where each intensity pixel corresponded to a unique coordinate location in the cell. These unique coordinates were matched to the plasma membrane, cytosolic, and nucleus compartments based on the location of DAPI and the CellMask plasma membrane stain, and the relative location of the eGFP-cavin-1 to these compartments was calculated.

### Immunostaining and particle analysis

Cells were grown to ∼75% confluency and exposed to stress conditions, then fixed with 3.7% formaldehyde, permeabilized using 0.2% Triton X-100 in PBS, and then blocked using 100% FBS. Cells were then stained with primary antibodies incubated for 2 h at room temperature or overnight at 4 °C, washed, and treated with a fluorescent secondary antibody for 1 h. Some of the primary antibodies that were used were anti-PABPC1 (Santa Cruz, sc-32318), antiG3BP1 (Santa Cruz, sc-81940), and anti-LSM14A (ABclonal Cat No: A16682). After another wash, the 35-mm MatTek glass bottom culture dishes were imaged on the ISS Alba FLIM 2-photon confocal microscope using a 100X/1.49 oil TIRF objective to microscopically count the number of particles formed under different conditions per μm^2^. For each condition, 10 to 12 cells were randomly selected, and z-stack measurements were taken (1.0 μ/frame). Analysis was performed using ImageJ where each measurement was thresholded before averaging the number of particles per frame per measurement. Statistical significance was calculated using SigmaPlot with a Student’s *t* test, Tukey test, or ANOVA on Ranks.

### N&B measurements

N&B theory and measurement has been fully described (refer to the study by Digman et al (29)). Experimentally, we collected ∼100 cell images viewing either free eGFP (control) or eGFP-Ago2, at a 66-nm/pixel resolution and at a rate of 4 μs/pixel. Regions of interest (256 × 256 box) were analyzed from a 320 × 320 pixel image. Offset and noise were determined from the histograms of the dark counts performed every two measurements. N&B data were analyzed using SimFC (www.lfd.uci.edu).

### N&B analysis

N&B defines the number of photons associated with a diffusing species by analyzing the variation of the fluorescence intensity in each pixel in the cell image. In this analysis, the apparent brightness, B, in each pixel is defined as the ratio of the variance, σ, over the average fluorescence intensity <k>:

BB=σσ2/<kk>. and<kk>=∈∈∈∈

where n is the number of fluorophores. The determination of the variance in each pixel is obtained by rescanning the cell image for ∼100 times as described earlier. The average fluorescence intensity, <k>, is directly related to the molecular brightness, €, in units of photons per second per molecule, and n. B can also be expressed as

BB =∈∈+1

And the apparent number of molecules, N, as

NN =∈∈∈∈/(∈∈+1)

### Western blotting

Samples were placed in six-well plates and collected in 250 μl of lysis buffer that included NP-40 and protease inhibitors as mentioned earlier, and sample buffer is added at 20% of the total volume. After SDS-PAGE, protein bands were transferred to nitrocellulose membrane (Bio-Rad). Primary antibodies include anti-PLCβ1 (Santa Cruz, sc-5291), anti-Ago2 (Abcam, ab32381), anti-actin (Abcam, ab8226), and anti-eGFP (Santa Cruz, sc-8334). Membranes were treated with antibodies diluted 1:1000 in 0.5% milk, washed 3 times for 5 min, before applying secondary antibiotic (anti-mouse or anti-rabbit from Santa Cruz) at a concentration of 1:2000. Membranes were washed 3 times for 10 min before imaging on a Bio-Rad chemi-doc imager to determine the band intensities. Bands were measured at several sensitivities and exposure times to ensure the intensities were in a linear range. Data were analyzed using ImageJ.

## Data availability

The authors will freely share all primary and analyzed data upon request. Please contact the corresponding author.

## Supporting information

This article contains supporting information.

## Conflict of interest

The authors declare that they have no conflicts of interest with the contents of this article.

## Figures and Tables

**S2A.jpg:**
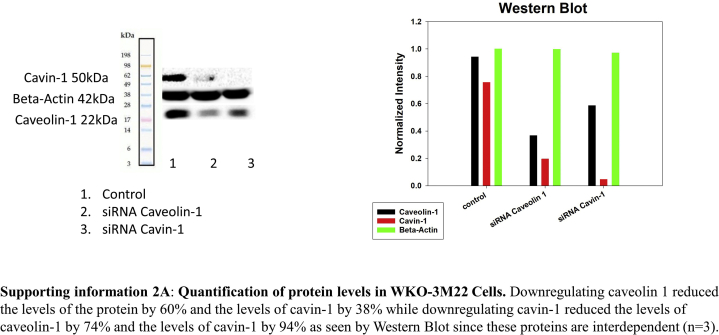


**S2B.jpg:**
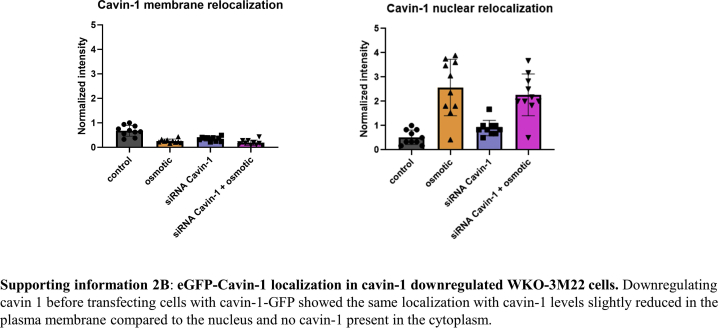


**S2C.Jpg:**
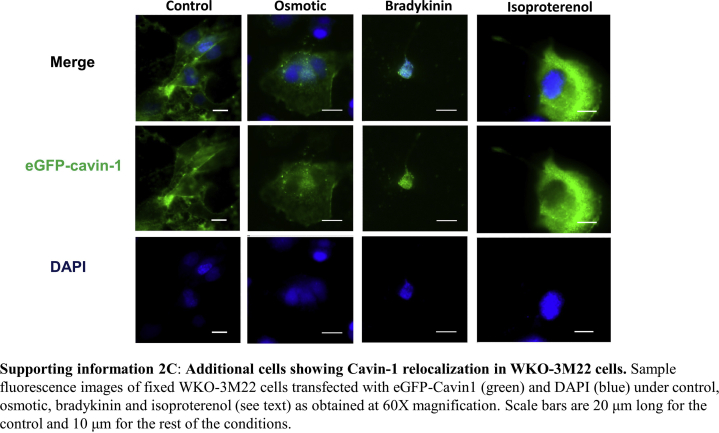


**SFD; IMAGE:**
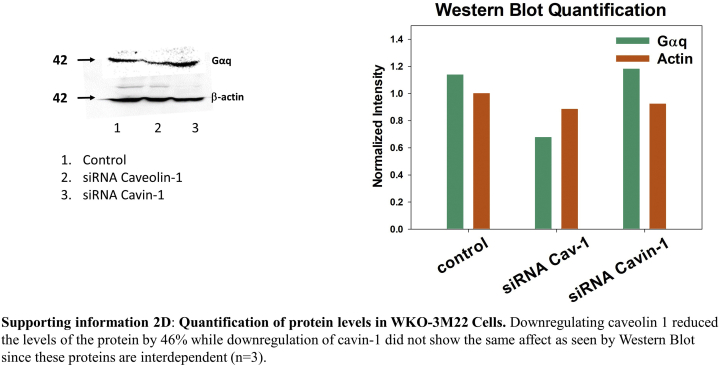


**SED.JPG:**
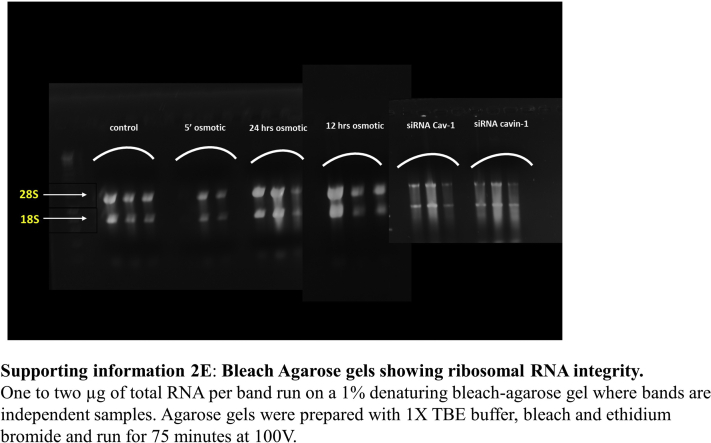


**S2F.JPG:**
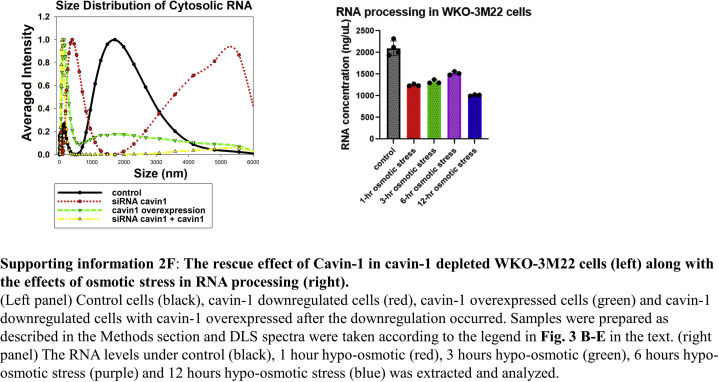


**S2G.JPG:**
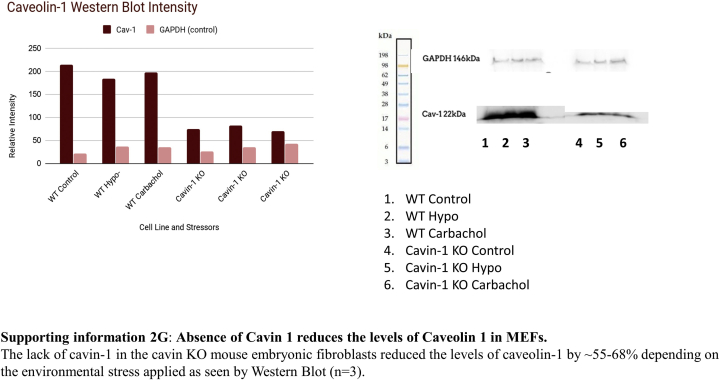


**S3.JPG:**
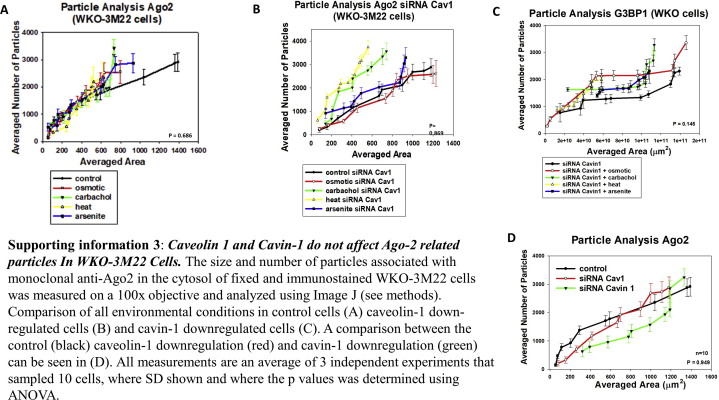


**S4.JPG:**
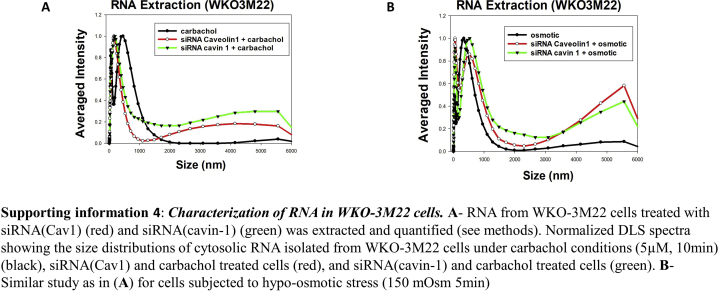


**S, S5.JPG:**
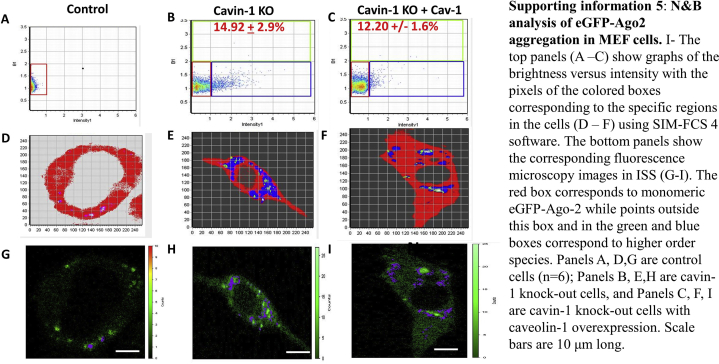


**S6; IMAGE:**
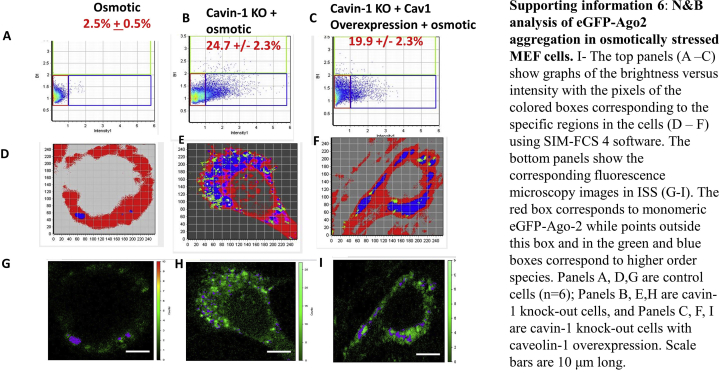


**SF1.JPG:**