# Supplementary figures and images for: Deformation of caveolae impacts global transcription and translation processes through relocalization of cavin-1
Source: J Biol Chem. 2022 May 2;298(6):102005. doi: 10.1016/j.jbc.2022.102005 (PMC9168624; doi:10.1016/j.jbc.2022.102005)

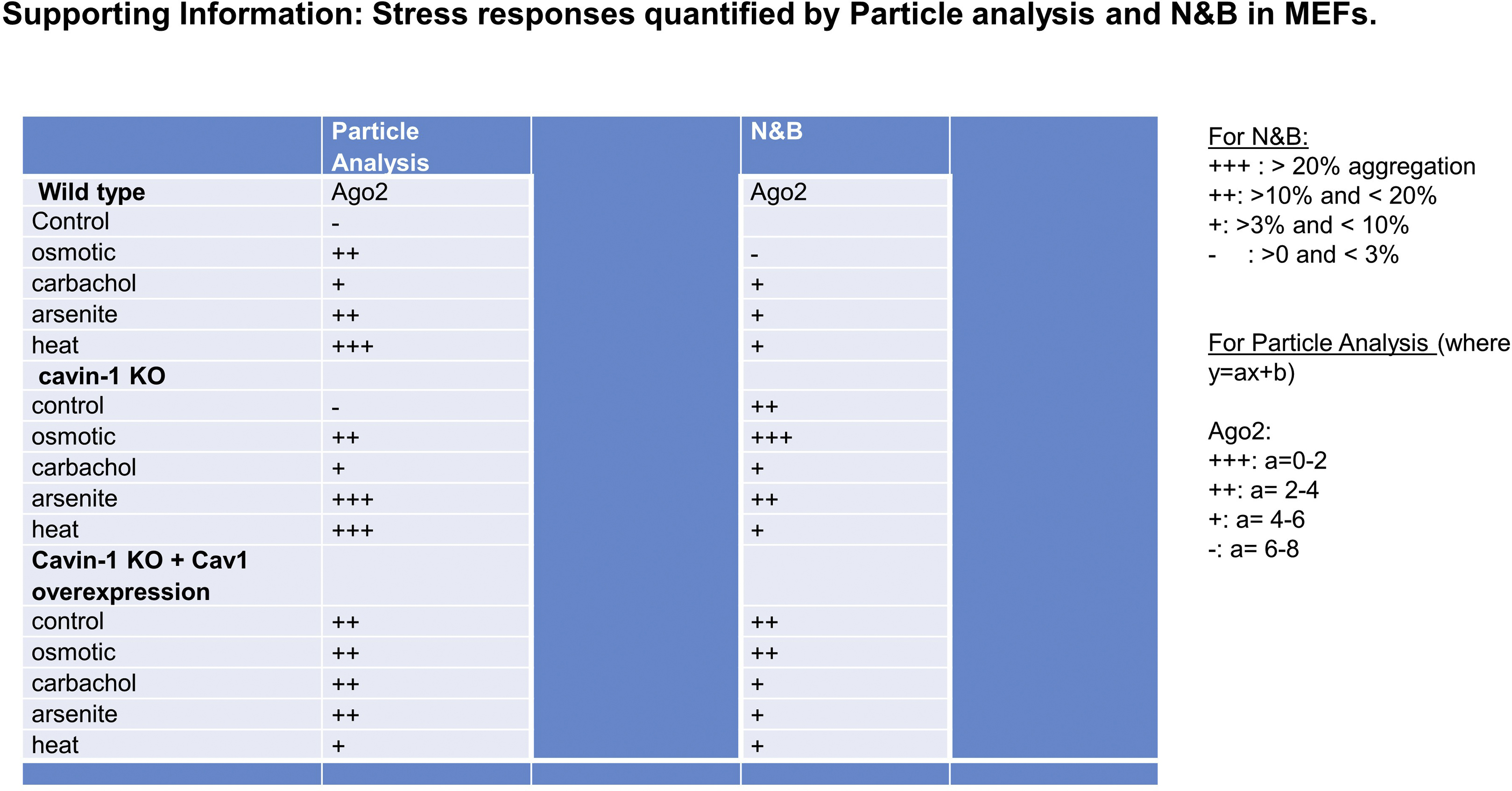

Supplement: S table [file figs13.jpg]
